# P-2240. Metabolomic Approaches to Detect Implant-Associated Infections in Post-Mastectomy Women

**DOI:** 10.1093/ofid/ofae631.2393

**Published:** 2025-01-29

**Authors:** John A Wildenthal, Margaret A A Olsen, Hung Tran, John I Robinson, David K Warren, Jeffrey P Henderson

**Affiliations:** Washington University in St. Louis School of Medicine, St. Louis, Missouri; Washington University School of Medicine, St. Louis, Missouri; Washington University, St. Louis, Missouri; Washington University in Saint Louis, Saint Louis, Missouri; Washington University School of Medicine in St. Louis, Saint Louis, Missouri; Washington University School of Medicine, St. Louis, Missouri

## Abstract

**Background:**

This year, approximately 54,000 women with breast cancer will undergo mastectomy followed by implant-based immediate breast reconstruction. This procedure has high infection rates of up to 20% and a diverse microbiology of infection including both gram-positive and gram-negative organisms. Culture-based diagnostic methods after these procedures suffer from poor sensitivity due to antibiotic administration prior to cultures, infection with fastidious organisms, and sequestration of bacteria in implant-associated biofilms. Culture-independent diagnostic methods may serve as an alternative for fast, more accurate diagnosis of these infections.

**Methods:**

Women undergoing implant-based immediate breast reconstruction were recruited. Seroma fluid was collected from the breast pocket at the time of implant removal due to infectious complications, non-infectious wound complications, or at the time of second stage reconstruction. Samples were analyzed using untargeted liquid chromatography/mass spectrometry (LCMS). Differential Compositional Variation Machine Learning (DiCoVarML) was used to discover metabolic features to differentiate infection status in this population.

**Results:**

80 seroma fluid samples taken at time of implant removal were analyzed. Of these, 37 samples were collected at the time of implant removal due to infection, 7 for non-infectious complications, and 36 were collected at the time of second stage reconstruction. After LCMS, 3663 high-quality features were detected in at least ten samples. Features useful in distinguishing infection status were selected using DiCoVarML. Predictive models were able to reliably distinguish infection status, with an area under the receiver operating characteristic curve of ∼0.85. Many features positively correlated with infection are structurally similar to peptides.

**Conclusion:**

Metabolite-based diagnostic methods appear to be promising tools to diagnose infection in immediate breast reconstruction. Features associated with infection may represent small immune proteins/peptides or degradation products from host or pathogen proteases. Further analysis is ongoing to fully identify metabolites associated with infection.

**Disclosures:**

Margaret A A. Olsen, PhD, MPH, Pfizer: Advisor/Consultant|Rebyota: Advisor/Consultant

